# Characterization of antibiotic prescription in intensive care units according to the Access, Watch, and Reserve classification

**DOI:** 10.15649/cuidarte.4340

**Published:** 2025-07-11

**Authors:** Carlos Augusto Solórzano, Edgar Fabián Manrique-Hernández, Angela Miranda Barajas, María Alejandra Caro, María Camila Rubio, Maricel Licht-Ardila, Alexandra Hurtado-Ortiz

**Affiliations:** 1 Fundación Cardiovascular de Colombia, Piedecuesta, Santander, Colombia. E-mail: carlossolorzano@fcv.org Fundación Cardiovascular de Colombia Santander Colombia carlossolorzano@fcv.org; 2 Fundación Cardiovascular de Colombia, Piedecuesta, Santander, Colombia. Public health department. Universidad Industrial de Santander, Colombia. E-mail: fabianmh1993@gmail.com Fundación Cardiovascular de Colombia Santander Colombia fabianmh1993@gmail.com; 3 Fundación Cardiovascular de Colombia, Piedecuesta, Santander, Colombia. E-mail: angelammirandab@gmail.com Fundación Cardiovascular de Colombia Santander Colombia angelammirandab@gmail.com; 4 Fundación Cardiovascular de Colombia, Piedecuesta, Santander, Colombia. E-mail: mariacaro@fcv.org Fundación Cardiovascular de Colombia Santander Colombia mariacaro@fcv.org; 5 Fundación Cardiovascular de Colombia, Piedecuesta, Santander, Colombia. E-mail: marcamilarubio240@gmail.com Fundación Cardiovascular de Colombia Santander Colombia marcamilarubio240@gmail.com; 6 Fundación Cardiovascular de Colombia, Piedecuesta, Santander, Colombia. E-mail: mlichtart@gmail.com Fundación Cardiovascular de Colombia Santander Colombia mlichtart@gmail.com; 7 Fundación Cardiovascular de Colombia, Piedecuesta, Santander, Colombia. Postgraduate Department in Infectious Disease, Universidad de Santander, Santander, Colombia. E-mail: alexandrahurtado@fcv.org Fundación Cardiovascular de Colombia Santander Colombia alexandrahurtado@fcv.org

**Keywords:** Antibiotic, Antibiotic Prophylaxis;, Drug Prescription, Infection, Drug Resistance, Microbial, Antibiótico, Profilaxis Antibiótica, Prescripción de Medicamentos, Infección, Farmacorresistencia Microbiana, Antibiótico, Antibioticoprofilaxia, Prescrição de Medicamentos;, Infeção, Resistência Microbiana a Medicamentos

## Abstract

**Introduction:**

The inappropriate use of antibiotics in intensive care units poses risks, such as increased infections caused by multidrug-resistant bacteria and adverse reactions. The World Health Organization's strategy, named Access, Watch, and Reserve, aims to mitigate these risks by categorizing antibiotics into these categories.

**Objective:**

To characterize antibiotic consumption in the adult population of intensive care units during the first quarter of 2023.

**Materials and Methods:**

A cross-sectional study on patients in intensive care units was conducted. A bivariate and multivariate analyses with logistic regression were carried out.

**Results:**

807 intensive care unit patients were studied, with a median age of 60 years. Piperacillin/tazobactam was the most prescribed antibiotic. According to the Access, Watch, and Reserve classification, 77.96% of prescriptions fell into Watch category, 11.29% into Reserve, and 10.75% into Access.

**Discussion:**

Antibiotic use in intensive care units is crucial for managing critically ill patients. Our study focuses on the challenges of antibiotic selection, complication management, and emphasizes antimicrobial stewardship for optimal therapy and reduced resistance.

**Conclusion:**

It is crucial to conduct an intervention study to demonstrate how increasing interaction of the antimicrobial stewardship team during prescription can enhance antibiotic use, reduce side effects, and decrease unnecessary costs.

## Resumen

**Introducción:**

El uso inapropiado de antibióticos en unidades de cuidados intensivos conlleva riesgos como el aumento de infecciones causadas por bacterias multiresistentes y reacciones adversas. La estrategia de la Organización Mundial de la Salud, denominada Acceso, Vigilancia y Reserva, tiene como objetivo mitigar estos riesgos al clasificar los antibióticos en estas categorías.

**Objetivo:**

Caracterizar el consumo de antibióticos en la población adulta de las unidades de cuidados intensivos durante el primer trimestre de 2023.

**Materiales y métodos:**

Se realizó un estudio transversal de pacientes en unidades de cuidados intensivos. Se llevó a cabo un análisis bivariado y multivariado con regresión logística.

**Resultados:**

Se estudiaron 807 pacientes en unidades de cuidados intensivos, con una edad media de 60 años. El antibiótico más prescrito fue Piperacilina/Tazobactam. En la clasificación de Acceso, Vigilancia y Reserva, el 77.96% de las prescripciones fueron Vigilancia, el 11.29% Reserva y el 10.75% Acceso.

**Discusión:**

El uso de antibióticos en las unidades de cuidados intensivos es crucial para el manejo de pacientes críticamente enfermos. Nuestro estudio se centra en los desafíos de la selección de antibióticos, el manejo de complicaciones y enfatiza la administración para una terapia óptima y una reducción de la resistencia.

**Conclusiones:**

Es crucial realizar un estudio de intervención que demuestre cómo aumentar la interacción entre el equipo de administración de antimicrobianos durante la prescripción puede mejorar su uso, reducir los efectos secundarios y disminuir los costos innecesarios.

## Resumo

**Introdução:**

O uso inadequado de antibióticos em unidades de terapia intensiva traz riscos como aumento de infecções causadas por bactérias multirresistentes e reações adversas. A estratégia da Organização Mundial da Saúde, denominada Acesso, Vigilância e Reserva, visa mitigar esses riscos categorizando os antibióticos nessas categorias.

**Objetivo:**

Caracterizar o consumo de antibióticos na população adulta de unidades de terapia intensiva durante o primeiro trimestre de 2023.

**Materiais e métodos:**

Foi realizado um estudo transversal em pacientes em unidades de terapia intensiva. Foi realizada uma análise bivariada e multivariada com regressão logística.

**Resultados:**

Foram estudados 807 pacientes de unidade de terapia intensiva, com mediana de idade de 60 anos. O antibiótico mais prescrito foi Piperacilina/Tazobactam. Na classificação de Acesso, Vigilância e Reserva, 77,96% das prescrições foram Vigilância, 11,29% Reserva e 10,75% Acesso.

**Discussão:**

O uso de antibióticos em unidades de terapia intensiva é crucial para o tratamento de pacientes gravemente doentes. Nosso estudo se concentra nos desafios da seleção de antibióticos, no tratamento de complicações e enfatiza a administração para terapia ideal e resistência reduzida.

**Conclusões:**

É crucial conduzir um estudo de intervenção demonstrando como o aumento da interação entre a equipe de administração antimicrobiana durante a prescrição pode aumentar seu uso, reduzir efeitos colaterais e diminuir custos desnecessários.

## Introduction

Antibiotics are among the most commonly used medications in intensive care units (ICUs), posing a high likelihood of inappropriate and excessive use^1^,^2^. Moreover, a higher proportion of resistant bacteria has been observed in environments with high antibiotic density, such as ICUs^3^,^4^. A study in China, involving 454 patients, demonstrated that antibiotic use promoted infections by resistant microorganisms increasing from 3% to 30%. The use of more than two antibiotics raises this range from 6.7% to 27%^5^. Similarly, research conducted in a Turkish ICU revealed that the percentage of inappropriate antibiotic use for Gram positive bacteria reached 83%, along with a low proportion of therapeutic de-escalation and inadequate antibiotic dose adjustments according to glomerular filtration rate^6^. 

In addition to bacterial resistance, the inappropriate use of antibiotics can lead to adverse reactions that significantly impact morbidity and mortality. *Clostridioides difficile infection* (CDI), although not usually associated with resistance, is closely linked to antibiotic misuse and can result in severe, potentially fatal diarrhea. According to the 2019 report by the Centers for Disease Control and Prevention (CDC), more than 2.8 million antimicrobial-resistant infections occur annually in the United States, resulting in over 35,000 deaths. When C. difficile infections are included, the total number of infections exceeds 3 million, with approximately 48,000 deaths associated with this resistant threat^7^. In environments such as ICUs, where patients often have multiple risk factors, inappropriate antibiotic use increases the incidence of CDI-related diarrhea. Moreover, improper prescription can lead to serious adverse effects in patients following repeated administration of these antibiotic medications^8^. 

The National Institute of Health of Colombia published data on antibiotic consumption trends from2015 to 2020, revealing that in ICU services, the antibiotics with the highest Defined Daily Dose (DDD) were meropenem, followed by piperacillin-tazobactam, and vancomycin in third place^9^,^10^. 

Regarding the frequency of antibiotic consumption in ICUs at the national level for 2021, it was identified that ceftriaxone consumption increased compared to the previous year, rising from 8.5 to 9.2 DDD per 100 bed-days^9^. Other monitored antibiotics showed a decrease: ertapenem from 3.7 to 0.5 DDD, cefepime from 9.4 to 7.6 DDD, piperacillin-tazobactam from 17.9 to 14.4 DDD, vancomycin from 13.9 to 12.2 DDD, and meropenem from 19.4 to 18.1 DDD^11^. 

A study published in 2021 examined the trend of antimicrobial resistance (AMR) and antibiotic consumption across 52 ICUs in Switzerland from 2009 to 2018. It was found that penicillins, cephalosporins, and carbapenems were the three most frequently used antibiotic groups. Over the study period, piperacillin/tazobactam consumption increased from 8.0 to 11.0 DDD per 100 bed-days (p=0.003), and ceftriaxone from 6.3 to 7.9 DDD per 100 bed-days (p<0.001). Meropenem use varied according to the geographic region, with higher consumption observed in the eastern and southeastern regions of Switzerland (11.4 vs. 15.6 DDD/100 bed-days; p=0.002). Quinolone consumption also increased from 3.0 to 4.0 DDD per 100 bed-days (p=0.014)^12^. 

Among the existing initiatives to control the indiscriminate use of antibiotics is the AWaRe strategy (Access, Watch, and Reserve)^13^ proposed by the World Health Organization (WHO). This strategy entails a classification system for antimicrobial use into three categories to facilitate antibiotic selection and minimize the risk of resistance development. Access and Watch categories include first and second-line treatment options, while the Reserve group comprises antibiotics indicated only for infections caused by multidrug-resistant (MDR) organisms, requiring strict monitoring and control^14^. The impact of a pharmacist-led antibiotic stewardship program in a pediatric ICU showed a 64% reduction in antibiotic use and a 58% reduction in healthcare costs^10^. 

Given these considerations, the importance of investigating antibiotic use in ICUs becomes evident. In this context, the main objective of this study is to characterize antibiotic consumption in the adult ICU population during the first quarter of 2023. 

## Materials and Methods

An analytical cross-sectional study was conducted on patients admitted to the ICU at a high-complexity institution in the northeastern region of the country during the first semester of 2023. The study included adult patients aged 18 and older, admitted to the ICU for more than 24 hours, and who received at least one dose of an antibiotic. The data collected in its entirety is available for free access and consultation in Zenodo^15^. 

The study included sociodemographic variables such as age, sex, health insurance scheme (contributory, subsidized, and special), as well as clinical variables, including comorbidities (diabetes, hypertension, cardiovascular disease, cerebrovascular disease, chronic kidney disease, rheumatological disease, hematologic cancer, solid organ cancer, benign prostatic hyperplasia, and chronic obstructive pulmonary disease). Other clinical variables included type of infection (meningitis, cerebral abscess, moderate pneumonia, severe pneumonia, complicated pneumonia, uncomplicated intra-abdominal infection, complicated intra-abdominal infection, lower urinary tract infection, upper urinary tract infection, cellulitis, erysipelas, necrotizing fasciitis, pyomyositis, osteomyelitis, periprosthetic joint infection, bacteremia, and head and neck infection), infectious diseases consultation, ICU length of stay, in-hospital mortality, and AWaRe categorization. 

The antimicrobials evaluated were: Ampicillin Sulbactam 1.5g, Oxacillin 1g, Ampicillin sodium 1g, Trimethoprim + sulfamethoxazole 80mg + 400mg, Meropenem 1g, Piperacillin 4g + Tazobactam 0.5g, Clindamycin 600mg/4mL, Vancomycin hydrochloride 500mg, Cephadroxil 1g, Cefazolin 1g, Penicillin sodium 5,000,000IU, Cefepime 1g, Avibactam 500mg + Ceftazidime 2000mg, Linezolid 2mg, Ertapenem 1g, Daptomycin 500 mg, Amikacin 500mg, Ceftriaxone 1g, Clarithromycin 500mg, Gentamicin 160mg, Tigecycline 50mg, Aztreonam 1g, Ceftaroline 600mg, Ciprofloxacin 100 mg. 

For the statistical analysis, normality was assessed using the Kolmogorov-Smirnov test. Measures of central tendency (mean, median) and dispersion (standard deviation or interquartile ranges) were calculated for continuous variables, according to their distribution. Categorical variables were described using frequencies and percentages. Bivariate analysis was performed with AWaRe classification as the outcome, using the chi-square test for categorical variables, Student's t-test for continuous variables with normal distribution, and the Mann-Whitney U test for non-normally distributed variables. Additionally, a multivariate logistic regression model was applied for each AWaRe category (Access, Watch, and Reserve), using Stata 16 software. 

**Ethical considerations**

This study was submitted to and approved by the Institutional Ethics Committee of the Fundación Cardiovascular de Colombia under resolution number CEI-2024-07306, on March 1, 2024, in accordance with national and international ethical guidelines for scientific research. Ethical principles and privacy regulations regarding patient data management were strictly followed to ensure the protection of confidentiality and sensitive information. All analyzed data were anonymized and remain under the custody of the FCV. 

## Results

A total of 807 ICU patients were analyzed during the study period. The median age was 60 years (IQR 45-72), and 53.78% were male. Regarding healthcare insurance, most patients (55.76%) were affiliated with the subsidized scheme, followed by the contributory scheme (32.09%). Infectious disease consultations were conducted for 38.25% of patients during their ICU stay. In terms of antibiotic exposure, 37.17% of patients received three or more antibiotics, 32.47% received two, while 30.36% received only one antibiotic. Hypertension was the most common comorbidity (38.41%), followed by solid organ cancer (24.16%), diabetes (22.68%), cardiovascular disease (18.09%), chronic kidney disease (10.90%), and cerebrovascular disease (8.92%). Additionally, cultures were obtained from 58.12% of patients not receiving prophylactic treatment, and the overall in-hospital mortality rate was 22.76% ( Table 1).

**Table 1:** General characteristics of patients treated in the ICU during the first semester of 2023

Variable	n	%
Sex		
Female	373	46.22
Male	434	53.78
Age**	60 (45-72)	
Health insurance		
Subsidized	450	55.76
Contributory	259	32.09
Special, exception, and others	98	12.15
Infectious diseases consultation*	210	38.25
Number of antibiotics received**	2 (1-3)	
Number of antibiotics categorized		
1	245	30.36
2	262	32.47
≥ 3	300	37.17
Comorbidities	631	78.19
Hypertension	310	38.41
Diabetes	183	22.68
Cardiovascular Disease	146	18.09
Stroke	72	8.92
Chronic Kidney Disease	88	10.90
Rheumatologic Disease	15	1.86
Hematologic Cancer	33	04.09
Solid Organ Cancer	195	24.16
Solid Organ Transplant	4	0.50
HSCT	1	0.12
COPD	69	8.55
HIV	7	0.87
Cirrhosis	12	1.49
ICU length of stay (days)**	4 (2-8)	
Laboratory culture sampling*	469	58.12
In-hospital mortality	181	22.76

*The patients receiving antibiotic prophylaxis were not considered. **median (IQR: interquartile range) HSCT:Hematopoietic Stem Cell Transplantation, COPD: Chronic Obstructive Pulmonary Disease, HIV: Human Immunodeficiency Virus.

When evaluating the indication or diagnosis for which antibiotics were prescribed, 32% of cases were for prophylaxis, followed by moderate pneumonia (14.88%) and uncomplicated intra-abdominal infection (12.89%). Other less frequent diagnoses include unspecified sepsis (8.30%), tracheobronchitis (7.93%), and lower urinary tract infection (5.33%). Bacteremia was present in 9.79% of the patients. The data revealed significant associations between various diagnoses and the number of antibiotics administered; pneumonia, intra-abdominal infection, cellulitis, osteomyelitis, bacteremia, and tracheobronchitis were significantly associated with antibiotic prescription (p <0.001), as shown in Table 2.

**Table 2:** Infectious diagnoses and number of prescribed antibiotics in the ICU during the first semester of 2023

Diagnosis	Patients		Number of antibiotics					p-value
	n	%	25 p	Median	75 p	Mean	SD	
Meningitis	20	2.48	2	3.5	5	2.41	2.41	0.001
Cerebral abscess	7	0.87	2	3	5	3.71	1.60	0.022
Infection of the head and neck	4	0.50	1.5	2	3	2.25	1.25	0.897
Pneumonia								<0.001
Moderate	120	14.88	2	2	4	2.83	1.60	
Severe	27	3.35	2	3	4	3.29	2.09	
Complicated	2	0.25	2	2.5	3	2.5	0.71	
Intra-abdominal infection								<0.001
Uncomplicated	104	12.89	2	3	4	2.90	1.41	
Complicated	21	2.6	2	2	4	2.90	1.64	
Urinary tract infection								0.803
Lower	43	5.33	1	2	3	2.55	1.67	
Upper	15	1.86	1	2	4	2.53	1.62	
Cellulitis	30	3.72	2	3.5	5	3.5	1.79	0.003
Necrotizing fasciitis	4	0.50	4	5	6.5	5.25	1.5	0.003
Pyomyositis	4	0.50	1.5	2	7	4.25	5.1	0.881
Osteomyelitis	25	3.10	3	4	5	4.44	2.58	<0.001
Periprosthetic infection	1	0.12	3	3	3	3.00	-	0.447
Bacteremia	79	9.79	2	3	5	3.56	1.89	<0.001
Fungemia	5	0.62	3	4	5	4.4	1.67	0.007
Tracheobronchitis	64	7.93	2	3	4	2.79	1.39	0.010
Antimicrobial prophylaxis	258	32	1	2	2	1.75	0.99	<0.001
Unspecified sepsis	67	8.30	1	2	3	2.22	1.20	0.357

25 p: 25th percentile, 75 p: 75th percentile

In total, 23 different antibiotics were identified as being administered to the studied patients during their ICU stay. Piperacillin/Tazobactam was the most frequently prescribed antibiotic, accounting for 64.3% of cases. The median units administered per day were 3.75 (IQR 3 - 4.12), corresponding to an average daily dose of 17 grams. Similarly, Meropenem was prescribed in 42.99% of cases, with a median of 22 units administered and a median treatment duration of 8 days. The average number of units administered per day was 3 (IQR 2 to 3.4), with a daily dose of 3 grams. Ampicillin Sulbactam was prescribed in 35.7% of patients, with a median of 21.5 units administered over a median of 3 days (IQR 1-7). The median units administered per day were 7.59 units, with a daily dose of 33 grams. Other antibiotics were used in lower proportions, as shown in Supplementary Table 1. 

The most utilized antibiotic group was the β-lactams, prescribed to 99.13% of the patients included in the study. Glycopeptides were the second most frequently used, administered to 25.03% of the patients, followed by oxazolidinones to 8.84% of the patients. Sulfonamides and aminoglycosides were utilized in 6.94% and 6.57% of the patients, respectively. Additionally, Macrolides and lincosamides were each prescribed in 4.34% of cases, while monobactams were used in 1.36%, lipopeptides in 0.74%, quinolones in 0.37%, and tetracyclines in 0.37%. 

**Figure 1:**
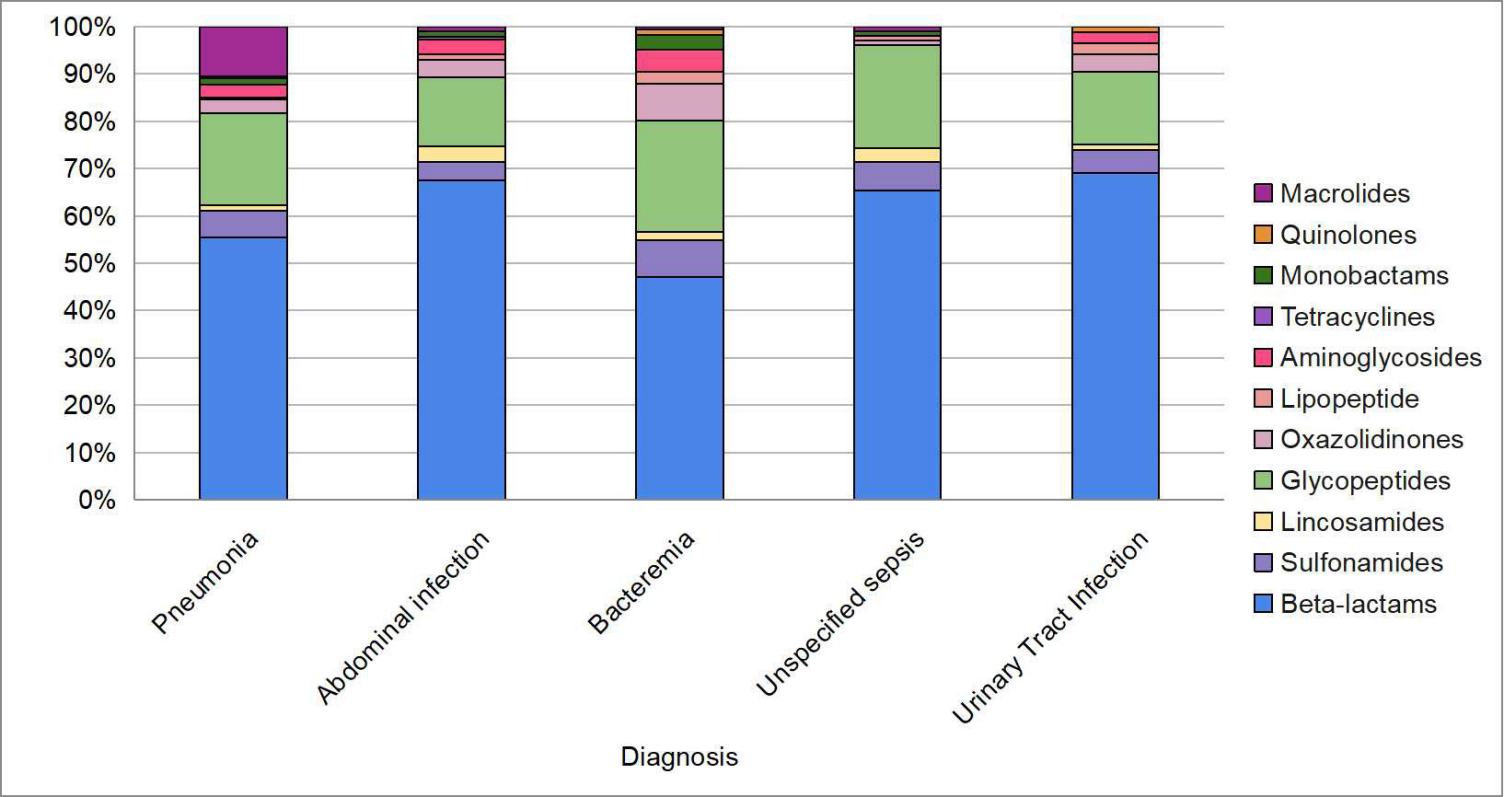
Antibiotic Usage by Most Common Types of Infection

As for the most frequently used antibiotics by type of infection, it was found in this research that beta-lactams are the preferred group for the treatment of pneumonia (55%), followed by glycopeptides (19%) and macrolides (10%). Similarly, in the case of intra-abdominal infection, beta-lactams and glycopeptides remained the most commonly used, at 68% and 15%, respectively. This same pattern was observed for other types of infections (Figure 1). 

**Figure 2:**
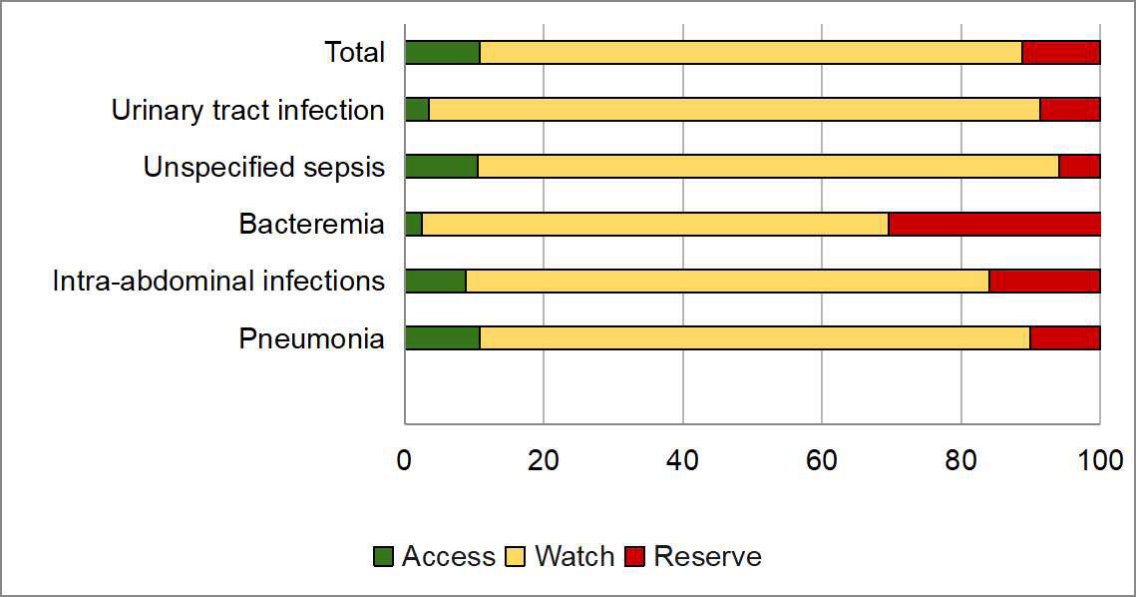
AWaRe Classification of Antibiotics According to the WHO

According to the AWaRe classification, the majority of antibiotics were categorized as Watch (77.96%), followed by Reserve (11.29%), and Access (10.75%). Similarly, the review indicated a comparable trend in antibiotic use by diagnosis within the Watch category. However, in patients with pneumonia and unspecified sepsis, Access category of antibiotics was the second most frequently used at 10.74% and 10.45%, respectively (Figure 2). Furthermore, infectious disease specialist consultations were conducted in 5.08% of patients receiving Access antibiotics, 33.88% of patients in the Watch group, and 100% in the Reserve group. 

A bivariate analysis was conducted between clinical and sociodemographic variables and the WHO's AWaRe classification of antibiotics. Of the total patients, 7.8% received Reserve antibiotics, 71.62% received Access antibiotics, and 68.15% received Watch antibiotics. No significant differences by sex were found in the Access and Watch categories (p = 0.081 and p = 0.429, respectively). Regarding age, the Reserve category showed a lower median age (54 years) compared to the other groups (60 years, p = 0.003). 

Significant differences were found in diagnoses across AWaRe groups. Regarding the access antibiotics administered to patients, significant differences were found for pneumonia (p=0.004), bacteremia (p=0.012), sepsis of unknown origin (p<0.001), and urinary tract infection (p<0.001). For Watch antibiotics, pneumonia showed a significant difference (p<0.001), as well as intra-abdominal infections (p<0.001), unspecified sepsis (p<0.001), and urinary tract infection (p<0.001). Finally, when analyzing Reserve antibiotics, a significant association was observed with intra-abdominal infections (p=0.001) and bacteremia (p<0.001). 

These results show that sex did not reach statistical significance in any of the antibiotic categories (Access, Watch, and Reserve). Regarding age, odd ratios (OR) were 0.98 (95% CI: 0.97-0.99, p= 0.021) and 0.97 (95% CI: 0.96-0.99, p=0.003) in the Watch and Reserve categories, respectively, suggesting that older individuals were less likely to receive antibiotics in these categories. 

Moderate and severe pneumonia had a higher likelihood of being treated with Watch antibiotics compared to the other groups, with ORs of 6.51 and 25.39, respectively (p <0.001 in both cases). Similarly, unspecified sepsis showed a higher likelihood of being treated with Watch antibiotics (OR 9.05, p <0.001), as well as lower urinary tract infection (OR 22.64, p<0.001). Regarding bacteremia, there was a higher likelihood of being prescribed with Reserve antibiotics (OR 8.85, p <0.001). As for intra-abdominal infection, there was a higher likelihood of being treated with Watch and Reserve antibiotics (Table 3). 

**Table 3:** Association between AWaRe classification and primary diagnoses

Variable	Access		Watch		Reserve	
	OR (95% CI)	p-value	OR (95% CI)	p-value	OR (95% CI)	p-value
Sex (Male)	1.39 (0.98; 1.89)	0.053	1.29 (0.92; 1.81)	0.129	1.23 (0.70; 2.16)	0.457
Age	0.99 (0.98; 1.00)	0.232	0.98 (0.97; 0.99)	0.021	0.97 (0.96; 0.99)	0.003
Pneumonia						
Moderate	0.39 (0.25; 0.60)	<0.001	6.51 (3.68; 11.49)	<0.001	-	-
Severe	0.64 (0.26; 1.55)	0.33	25.39 (3.39; 190.22)	0,001	-	-
Bacteremia	0.49 (0.29; 0.83)	0.01	-	-	6.85 (3.77; 12.45)	<0.001
Unspecified sepsis	0.21 (0.12; 0.36)	<0.001	9,05 (04.02; 20.37)	<0.001	-	-
Abdominal infection						
Uncomplicated	-	-	9.49 (4.87; 18.49)	<0.001	2.68 (1.35; 5.30)	0.005
Complicated	-	-	21.64 (2.89; 163.47)	0,00	3.68 (1.10; 12.24)	0.033
Urinary tract infection						
Lower	0.17 (0.08; 0.33)	<0.001	22.64 (5.35; 93.84)	<0.001	-	-
Upper	0.14 (0.04; 0.43)	0.001	-	-	-	-

For the variables Pneumonia, Abdominal infection, and Urinary tract infection, the reference category is defined as absence of infection.

## Discussion

The analysis of antibiotic use in ICUs is essential given the importance of these drugs in managing critically ill patients. As evidenced in various studies, inappropriate and excessive prescribing of antibiotics in these units presents a significant risk, exacerbating bacterial resistance and increasing the incidence of infections caused by resistant microorganisms. Our research focuses on a medical-surgical ICU, where patient population was predominantly older adults. Notably, half of the individuals were enrolled in the subsidized health insurance scheme. Additionally, the patients exhibited a wide range of comorbidities, with hypertension, diabetes mellitus, and solid organ neoplasms being the most prevalent. These preexisting conditions significantly increase the risk of complications during hospitalization, including susceptibility to infections^16^. 

Similarly, among the comorbidities, nearly 11% of patients had chronic kidney disease, which poses a significant challenge as it requires specialized attention in pharmacokinetics and pharmacodynamics for the appropriate selection and dosing of antibiotics. In our study, we observed that the use of antibiotics such as vancomycin, prescribed in one-quarter of cases, and amikacin carry a higher risk of nephrotoxicity. Conversely, beta-lactam antibiotics, used in nearly all cases, allow for dose adjustment, helping to reduce unwanted side effects such as acute kidney injury, cytopenias, neurotoxicity, among others^17^. 

Prophylaxis was the most common indication for antimicrobial use, and it is noteworthy that some of the antimicrobials used for this purpose fell into the Watch or Reserve categories. This situation presents a particularity as prophylactic prescriptions are protocol-based, and adherence is monitored retrospectively. Consequently, involvement of infectious disease specialists in prophylactic antibiotic decisions is not as active as in the case of infectious diseases. In most cases requiring prophylaxis, guidelines recommend the use of Access antibiotics, in line with the recommendations of Calderwood et al^18^. 

Pneumonia was the second leading cause for antimicrobial use and the most frequent among infectious pathologies. Following the onset of the SARS-CoV-2 pandemic, pneumonia has become a key focus in monitoring antibiotic exposure in ICUs. A significantly concerning outcome was that 90% of the antibiotics used in pneumonia cases fell into the Watch or Reserve categories, a proportion also observed in intra-abdominal infections. Therefore, surveillance of these groups of antibiotics should be increased. Considering that the AWaRe strategy recommends limiting Watch and Reserve antibiotic use to nor more than 40%, it is pertinent to implement strategies to reduce their consumption and increase the use of Access group antibiotics^13^. 

A study conducted by Waagsbø et al., which analyzed 1112 episodes of pneumonia, revealed that the initial use of broad-spectrum antibiotics occurred in 34.1% of cases, but decreased to 17.1% following the implementation of multiple interventions initiated in the emergency department. These interventions included educational activities on appropriate antibiotic use, microbiological sampling for analysis, therapy adjustments based on results, early transition to oral therapy, and adoption of shorter treatment regimens. Importantly, these changes did not lead to increased readmissions or mortality, suggesting a positive impact on adherence to clinical practice guidelines^19^. 

Regarding bacteremia, we found that it was the third condition with the highest Reserve antibiotic use in our cohort. It is crucial to highlight that the use of these drugs was primarily linked to immunocompromised patients, such as those with hemato-oncological disorders or human immunodeficiency virus infection, who often present with colonization or infection by multidrug-resistant microorganisms. This approach is supported by the implementation of detection strategies, such as screening for carbapenem-resistant Enterobacteriaceae^20^. In this context, agents like ceftazidime-avibactam, with or without aztreonam, are often prescribed, while linezolid and daptomycin are preferred alternatives in patients with renal dysfunction. 

Given that antimicrobial adjustment possibility depends on microbiological confirmation, the finding that samples for culture were not collected in 42% of cases suggests an ongoing gap between the diagnosis and treatment of infectious diseases in this ICU. The lowest rates of culture collection were seen in skin and soft tissue infections, followed by intra-abdominal infections and pneumonia. This scenario, in many cases, is associated with an inadequate strategy for microbiological study collection. This aspect requires a balance in sample collection, as it could also lead to unnecessarily antibiotic exposure. This aspect is crucial and becomes one of the cornerstones in the implementation of an Antimicrobial Stewardship Program (ASP)^21^,^22^. 

The number of patients with infectious disease therapy approval is less than 50%, emphasizing the need for an operational antimicrobial stewardship (AMS) team, led by the Infectious Disease service, to authorize all prescriptions of Watch and Reserve antibiotics. It is noteworthy that Infectious Disease service, according to the AWaRe classification, approved 100% of the Reserve antibiotics, while nearly 67% of the Watch antibiotics prescribed were not, highlighting a critical area for improvement. Mokrani et al., among other researchers, describe how ICU-based AMS teams can impact antibiotic consumption by reducing antimicrobial treatment duration, limiting the use of broad-spectrum antibiotics, avoiding anti-MRSA antibiotics, and restricting empirical and definitive therapy with combination regimens. The involvement of ICU physicians in AMS support teams has been shown to facilitate implementation and adherence to guidelines^23^,^24^. 

This study has some limitations. The evaluation period covered only one semester, suggesting the need for studies with longer durations and antimicrobial resistance assessments. Additionally, cohort studies are considered necessary to evaluate clinical outcomes and factors associated with specific events. 

## Conclusions

In conclusion, our findings highlight the high prevalence of antibiotic use in intensive care units (ICUs), with a considerable proportion of patients receiving multiple antimicrobial agents. Additionally, most prescribed antibiotics fall into the Watch category of the WHO's AWaRe classification, suggesting a cautious yet suboptimal use of these medications. This study addresses various aspects and variables influencing prescribing culture, emphasizing the need for targeted interventions to optimize their use. 

It is crucial to conduct an interventional study to demonstrate how increased interaction between the AMS team and the prescribing process can improve administration, reduce side effects, and potentially lower unnecessary costs. 

## Supplemental Material

**Supplementary Table 1:** Characteristics of the antibiotics administered in the ICU

A/B	No. (%) of patients	Total Quantity A/B (Units)	Days of A/B	Quantity per day by patients (Units)	Dosage
Piperacillin/tazobactam (g)	353 (64.3)	19 (9-32)	5 (2-9)	3.75 (3-4.125)	17 (13.5-18.56)
Meropenem (g)	236 (42.99)	22 (11.5-45)	8 (3.5-17)	3 (2-3.48)	3 (2-3.48)
Ampicillin+sulbactam (g)	196 (35.7)	21.5 (10-53)	3 (1-7)	7.59 (6-8.22)	32.5 (15-79.5)
Vancomycin (mg)	180 (32.79)	18 (6.5-50)	7 (2-19)	3.86 (2-4.79)	1933 (1000-2397.35)
Cefazolin (g)	118 (21.49)	3 (2-6)	1 (1-3)	2 (2-4)	2 (2-4)
Ceftriaxone (g)	91 (16.58)	10 (4-20)	4 (1-8)	2.5 (2-3.8)	3 (2.2-4)
Cefepime (g)	58 (10.56)	24.5 (15-42)	7 (5-10)	3.41 (2.9-5.42)	3 (2.9-5.42)
Trimethoprim-sulfamethoxazole (mg)	54 (9.84)	23.5 (7-124)	13 (3-35)	2.87 (0.5-8.732)	1380 (240-4186)
Ertapenem (g)	35 (6.38)	8 (4-12)	8 (3-12)	1.11 (1-1.16)	1 (1-1.16)
Clarithromycin (mg)	35 (6.38)	9 (4-14)	4 (2-7)	2.1 (2-2.25)	1050 (1000-1125)
Linezolid (mg)	31 (5.65)	28 (11-44)	14 (5-23)	2 (1.92-2.15)	4 (3.85-4.31)
Ceftazidime (g)	30 (5.46)	37.5 (20-59)	14(7-21)	2.92 (2.6-3)	7.31 (6.5-7.5)
Clindamycin (mg)	26 (4.74)	8 (1-17)	1.5 (1-5)	3.53 (1-4.14)	2120 (2485.71-1877.91)
Amikacin (mg)	23 (4.19)	7 (2-12)	4 (1-6)	2 (1-2.5)	2.4 (1.83-3)
Oxacillin (g)	17 (3.1)	70 (17-158)	7 (1-14)	11.26 (9.71-12.15)	11 (9.71-12.15)
Cephradine (g)	12 (2.19)	6.5 (4-16)	2 (1-4)	4 (3.12-4.1)	4 (3.12-4.1)
Aztreonam (g)	11 (2)	82 (24-124)	13 (4-20)	6 (4.1-6.2)	6 (4.1-6.2)
Daptomycin (mg)	6 (1.09)	31.5 (3-54)	35 (1-62)	1.08 (0.87-2)	544 (435.48-1000)
Gentamicin (mg)	5 (0.91)	5 (1-14)	4 (1-22)	1 (1-1.25)	160 (160-200)
Penicillin (IU)	4 (0.73)	23 (2.5-84.5)	4.5 (1.5-14)	4 (1.75-6.07)	20000000 (8750000-19600000)
Tigecycline (mg)	3 (0.55)	28 (19-61)	13 (9-31)	2.11 (1.96-2.15)	106 (98.38-107.87)
Ciprofloxacin (mg)	3 (0.55)	64 (8-233)	8 (2-29)	6400 (800-23300)	800 (400-803.44)
Ceftaroline (mg)	1 (0.18)	28 (N/A)	14 (N/A)	2 (N/A)	1200 (N/A)

Note: g=grams; mg=milligrams; IU=international units.
